# Urban health insurance reform and coverage in China using data from National Health Services Surveys in 1998 and 2003

**DOI:** 10.1186/1472-6963-7-37

**Published:** 2007-03-03

**Authors:** Ling Xu, Yan Wang, Charles D Collins, Shenglan Tang

**Affiliations:** 1 Centre of Health Statistics and Information, Ministry of Health, 1 Xizhimen Nanlu, Beijing, The People's Republic of China; 2 Liverpool School of Tropical Medicine, Pembroke Place, Liverpool, L3 5QA, UK; 3 Independent Academic, Liverpool, UK

## Abstract

**Background:**

In 1997 there was a major reform of the government run urban health insurance system in China. The principal aims of the reform were to widen coverage of health insurance for the urban employed and contain medical costs. Following this reform there has been a transition from the dual system of the Government Insurance Scheme (GIS) and Labour Insurance Scheme (LIS) to the new Urban Employee Basic Health Insurance Scheme (BHIS).

**Methods:**

This paper uses data from the National Health Services Surveys of 1998 and 2003 to examine the impact of the reform on population coverage. Particular attention is paid to coverage in terms of gender, age, employment status, and income levels. Following a description of the data between the two years, the paper will discuss the relationship between the insurance reform and the growing inequities in population coverage.

**Results:**

An examination of the data reveals a number of key points:

a) The overall coverage of the newly established scheme has decreased from 1998 to 2003.

b) The proportion of the urban population without any type of health insurance arrangement remained almost the same between 1998 and 2003 in spite of the aim of the 1997 reform to increase the population coverage.

c) Higher levels of participation in mainstream insurance schemes (i.e. GIS-LIS and BHIS) were identified among older age groups, males and high income groups. In some cases, the inequities in the system are increasing.

d) There has been an increase in coverage of the urban population by non-mainstream health insurance schemes, including non-commercial and commercial ones.

The paper discusses three important issues in relation to urban insurance coverage: institutional diversity in the forms of insurance, labour force policy and the non-mainstream forms of commercial and non-commercial forms of insurance.

**Conclusion:**

The paper concludes that the huge economic development and expansion has not resulted in a reduced disparity in health insurance coverage, and that limited cross-group subsidy and regional inequality is possible. Unless effective measures are taken, vulnerable groups such as women, low income groups, employees based on short-term contracts and rural-urban migrant workers may well be left out of sharing the social and economic development.

## Background

In 1997 a major reform of the government run urban health insurance system was undertaken in China. Previous to this, there were two government systems – the Government Insurance Scheme (GIS) and the Labour Insurance Scheme (LIS). Particular problems of these two systems were fragmentation, small risk pooling capacity and low population coverage. The absence of mechanisms to control 'moral hazard' behaviour by both suppliers and users also meant escalating costs. To address these problems, the Chinese government launched a nation-wide urban health insurance reform in 1997. The reform, which was based on local reforms during the 1990's [1], led to the establishment of a new "urban employee basic health insurance scheme" (BHIS). The new scheme expresses a shift towards a more cohesive policy framework and management. The aim of the reform was to widen coverage and the State Council set a target of 80 million employees to be covered through the new scheme by the year 2001[2]. By the end of 2004 BHIS covered more than 124 million people including employees and retirees, and 34.1% of the employed population in the urban areas [3].

It is well recognised that different forms and combinations of public and private health financing can have differing implications for equity through enhancing or impeding the access to and use of health services by different social groups. It has also been argued that the exact impact of different financing approaches on equity of access is yet to be fully understood [4]. As a form of financing, social health insurance has typically been used in countries experiencing the transition from planned to market economies [2]. It's establishment has been advocated by the World Health Organisation as a key to achieving universal coverage of health care and to protecting access to health services, particularly for the disadvantaged in less developed countries [5,6]. There is, however, considerable variety in the forms of social health insurance which, in turn, has an impact on access to and use of health services by different social groups. In those countries with established social insurance schemes there is continuing concern over the issues and challenges of fragmentation, partial population coverage and difficulties in generating sufficient pooling. The development of a fair, affordable and sustainable system of social health insurance represents a major challenge for health systems reformers.

Although the development of BHIS has been quick, it has not been without controversy and there are clearly important issues raised in the process of transition [1,2,7]. This paper focuses on a key area of controversy – coverage. The data from the National Health Services Surveys (NHSS) in 1998 and 2003 will be used to explore the impact of the reform on coverage. The importance of this paper resides in the insights provided by the NHSS. By the time of the 2003 survey, almost all the Chinese municipal governments had established BHIS in the urban cities. The data from the two surveys conducted in 1998 and 2003 can be a useful source in helping to interpret the impact of the 1997 reform. Analysis of the reform process in China also provides useful ideas for the development of comparative studies in health care financing.

Following this introduction, the paper will then describe the methods used by the NHSS. Data from the 1998 and 2003 surveys will then be compared in relation to overall coverage, and with reference to age, income level, employment status and gender. The discussion section then refers to three key explanatory features of coverage: organisational diversity, the labour market and its interface with the new insurance system, and regional diversity of non-mainstream health insurance schemes. The paper concludes by pointing to two areas of policy options open to the health system and possible features of follow-up research. However, before proceeding to the above subheadings, the background and the aims of the reform, and the new BHIS in China will be described.

Prior to 1997 there were two principal insurance schemes for the urban population in China – GIS and LIS. These had been established after the Chinese Communist Party came to power in 1949. Relying heavily on government funds, the urban health care system was established to cater for the health care needs of the urban elites and the labour force vital to the urgently needed industrial expansion. Both GIS and LIS were developed through central government policy guidelines although they were managed, funded and operated by local governments and enterprises for their own employees and retirees. In this sense, work units operated as "semi-enclosed communities" providing the constituencies with a package of free or heavily subsidised services including health care [2].

National industrialisation was a clear government priority and this had a propelling effect on the expansion of the urban labour workforce, leading to the rapid expansion of a complex and duplicated network of urban health care services [8]. As the proportion of funds allocated by government to health care providers fell from the 1980s onwards, health service providers turned to user fees to generate their income. In such circumstances the oversupply of services and drugs and the overuse of advanced clinical technology to insured patients became commonplace. The increase in the number of beneficiaries attached to both schemes, together with other factors such as the ageing of beneficiaries and the rapid surge of medical care costs, contributed to uncontrollable and unaffordable financial burdens on both governments and enterprises [1,9].

Another prominent issue was the inequality of health insurance coverage. This was expressed in two ways. Firstly, the entitlement for insurance benefits was determined by factors such as the workplace [10]. Whereas employees in state-owned enterprises were almost certainly covered by the insurance schemes, others might not. Secondly, individual enterprises varied in the services covered leading to differences in the financial burden of health care for employees in different enterprises [11].

**Figure 1:**
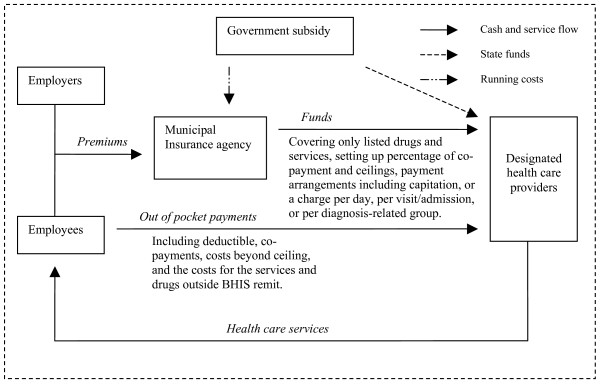
**The operation of BHIS system**. *Source: Based on Hsiao [12]*

The principal aims of the 1997 health insurance reform were: a) to achieve a wider population coverage with basic medical care benefits across all the groups participating in the scheme; and b) to control the oversupply and the over-utilisation of health care through setting mechanisms to alter the behaviour of health service users and providers [1]. The reform merged the two old systems – GIS and LIS – into a new insurance scheme known as Urban Employee Basic Health Insurance Scheme (BHIS). Figure 1 illustrates the operation of the new system in terms of cash flows and service provision together with the relationships between employers, employees, and government. Contributions are made by employers and employees to a municipal level insurance agency (the Department of Labour and Social Security at municipal or county level) which receives support from the government in terms of running costs. The agency sets out the lists of drugs and services covered by the scheme and the forms of provider payment. The providers, who also receive some state funding in the form of subsidies, receive payments from the municipal insurance agency and the insured employees.

Since the 1997 reform, there has been a process of transition from the old GIS-LIS system to the new BHIS, although both systems still operate in parallel. Certain key similarities and differences between the old and new systems may be highlighted.

### 1. Source of finance

In the GIS-LIS system only the government and individual enterprises make contributions to a fund. GIS is usually funded from local government budgets, while LIS is funded from welfare funds generated by enterprises. In the BHIS contributions are from employees (2% of annual wage/salary) and employers (up to 6% of employee's wage/salary although the employer's contribution can be as high as 10% as in the case in Shanghai).

### 2. Management

The GIS-LIS system is operated by individual local governments and enterprises with centrally determined rules and regulations. The new system is managed by insurance management agencies located within local governments. Within the BHIS national policy framework, local governments have the autonomy for setting up rules of collecting and allocating funds, and operating the scheme [7]. Therefore, payment mechanisms for out- and in-patient care may differ between cities.

### 3. Eligibility and beneficiaries

The GIS-LIS system is employment-based and mostly in public sector and state-and collectively-owned enterprises. The beneficiaries are the employees and the retirees, while dependents (children, spouses and/or parents who do not have proper employment related health insurance), have partial coverage. The BHIS system is employment based, covering both employees and retirees in the public sector and also extends to private and joint-venture enterprises. However, BHIS does not cover any dependents of the insured.

### 4. Payment arrangements

In the GIS-LIS there is an almost total refund for care provided. In the BHIS, premiums are calculated based on a percentage of individual income although the percentage may vary between cities. Deductibles, co-payment arrangements and ceilings have been established. The premium funds are divided into two tiers – the *Individual Account Fund *(IAF) and the *Social Pooling Fund *(SPF). The former is a pot of money made up of an income related contribution from the employee plus an age adjusted contribution from the employer. This means that the older the employee is, the greater proportion of the contribution from the employer is allocated into his/her individual saving fund. There is a yearly calculation of expenditure from the fund and under-spends can be carried forward to the next insurance year. The *Social Pooling Fund *is mainly from the employer's contribution and is used when the IAF has been depleted, and out-of-pocket expenditure (deductibles) has exceeded a pre-set percentage of annual income (e.g. 5% of annual income as in the case of Shanghai and Shenzhen). However, the SPF in most urban centres is only used to cover inpatient related expenditures. For using the SPF, co-payments between the insurance agency and the insured individual are established, although the co-payment percentage split between insurance agency and insured individual varies from city to city. A cost ceiling for using SPF is also set up, beyond which all medical costs are borne by the insured individual until next financial year. A few cities, like Shanghai, have developed so-called supplemental health insurance schemes which are designed to cover costs that go over the ceiling level.

### 5. Cost containment mechanisms

In the GIS-LIS system there is almost no restriction on the types of health care services and prescriptions provided. In the BHIS, health facilities are designated for use by the members. At the same time the insurance agency has also indicated the types of services and drugs that can be reimbursed/covered by the BHIS. Any services and drugs that are not listed are paid for by the individuals concerned. A fixed fee-for-service has also been introduced.

### 6. Enforcement of membership

In the GIS-LIS system, joining the schemes has been almost automatic for employees of an organisation linked to the insurance system. Although enterprises are required to participate in the BHIS, it was not made mandatory and some enterprises have chosen to stay away from the new scheme. Enterprises that are economically better-off and/or have a younger demographic profile among the employees have been more likely to stay out of BHIS, while there are some organisations that cannot afford the premiums [1,13]. For whatever the reasons, these enterprises may continue in GIS-LIS or develop their own insurance arrangements for employees. Insurance arrangements in the latter case may vary from one enterprise to another. Whatever the case, the decision to join BHIS or not is largely in the hands of the employer/owner and not the employees.

Although not directly related to the 1997 reform, other non-commercial and commercial insurance schemes have been established since the 1990s by both the public and private sectors to cover medical and health needs of the insured. For example, many insurance companies have worked with schools to develop schemes covering inpatient care for the children in primary and secondary schools in different cities. Private enterprises and companies, including joint ventures, have often tended to buy specific health insurance packages from these insurance companies. Overall, they tend to provide limited health care coverage for the insured and are complementary to the mainstream/principal insurance schemes such as GIS-LIS and BHIS. Although in-depth understanding of these health insurances schemes is presently lacking, it may be assumed that there is diversity in terms of the background of the insurance companies, as well as the nature and operation of the insurance schemes managed by them.

## Methods

Three National Health Services Surveys (NHSS) have been conducted in China for both urban and rural areas in June/July 1993, June/July 1998 and September/October 2003 (the postponement was due to SARS epidemic). These surveys were organised and directed by the Centre for Health Statistics and Information, Ministry of Health. This paper uses only the data generated from household questionnaire interviews carried out among the urban population in the 1998 and 2003 surveys. GIS-LIS and BHIS only operate in the urban areas and the BHIS only began to be implemented nationwide in 1998.

### Sampling strategy and methods

**Figure 2:**
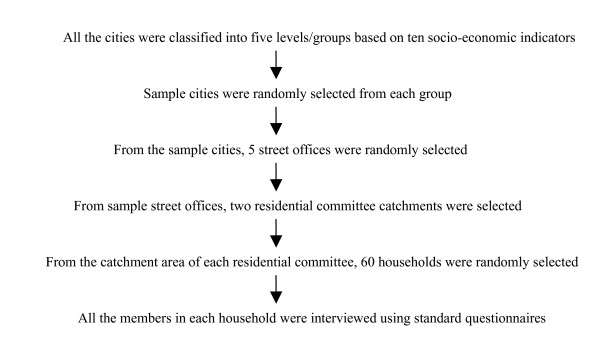
**NHSS sampling process**. *Source: Gao et al [15] and Centre for Health Statistics and Information [16]*

The survey sample was decided using multi-stage stratified random sampling procedures and methods in order to achieve maximum representation of the demographic and socio-economic characteristics of the whole population. All the cities in the country were grouped into 5 levels/groups according to 10 socio-economic, education, demographic and health indicators. These included employment rates in different types of industries, percentages of population under 14 and above 65, literacy rate, gross birth and death rates, infant mortality rate, per capita products of industry and agriculture and percentage of population with junior high school education. The cities ranked at the first level demonstrated higher social and economic development and the best health indicators, while those ranked at the fifth level demonstrated the opposite [14]. A stage by stage sampling process is illustrated in Figure 2, focusing only on urban areas although the same survey procedure was applied to both and rural areas as a whole:

All three NHSSs used the same sampling strategy and procedure. A comparison analysis test was conducted between the sample population and the data generated from the population census of 2000, confirming a high representation of the sample population in relation to the whole. The sample cities, households and population sizes in the surveys of 1998 and 2003 are presented in Table 1. In spite of slightly bigger household numbers, the smaller population sample in 2003 could be explained by the gradual decrease in average family size [17]. The average family size was 3.25 in 1998, and 2.96 in 2003 for urban areas [16,18].

**Table 1:** Sample sizes of NHSS, 1998 and 2003 (for urban areas)

Year	Cities	Households	Population
1998	28	16584	52287
2003	28	16811	49698

### Data collection

In the third NHSS, the households participating in the questionnaire interviews were randomly selected from the same sample residential committees that were used for the first and second NHSS [16]. The survey aimed to interview each member of the sample families and to collect the information directly from household members, except infants and young children whose information was collected through his/her mother/carer. When the scheduled respondent was absent (following several attempts to conduct the interview), one of the household members was asked to provide relevant information. There were 22.2% proxy respondents in 2003 NHSS (the proxy respondent rate was not calculated for 1998 NHSS). NHSS requested that the proxy respondent rate should be controlled at a figure below 30%. The interviewers were community level medical doctors who underwent prior training on conducting the household interviews. Supervision and quality inspection was conducted by medical doctors at a higher supervisory level.

### Data analysis

Analysis for this specific paper included data on coverage in relation to participation in health insurance, employment status, income level, age and gender. With respect to income groups, this paper has used the methods of Gao et al [15]. The total sample population is divided into five income groups/quintiles based on self-reported yearly income, each of which represents 20% of the total sample population, as shown in Table 2.

**Table 2:** Division of income groups

	Lowest	Lower	Middle	Higher	Highest
1998	less than ¥2040	¥2040–¥2999	¥3000–¥4169	¥4170–¥5999	more than 6000
2003	less than ¥2640	¥2640–¥4011)	¥4012–¥6059)	¥6060–¥9035	more than 9036

### Quality control

Quality control measures used in the NHSS included establishing a management structure and data reporting system, consultation meetings for the survey design and finalising survey tools, organising the training for the survey supervisors and interviewers and conducting pilot surveys.

Measures for the quality control of data collection included revisits to a sample of 5% of households by survey supervisors to check the accuracy of data collected by interviewers. During the second visit, fourteen key indicators/questions were asked to check the consistency of the information collected: being visited by an interviewer and duration of the visit, hospitalisation in the past one year, distance to the nearest health facilities, use of ambulance/emergency services, current monthly household income and expenditure. The range of matching rates of the key indicators between the first and second visits was 91–97%.

There were however important methodological issues to contend with. As the information collected by the NHSSs was self reported, there was an issue of recall accuracy of the information provided by the respondents. The use of proxy respondents in the absence of scheduled respondents may also challenge information accuracy. The use of local community doctors to conduct interviews meant that their knowledge of the local socio-economic, cultural and geographic context would facilitate the process of the survey. On the other hand, it might have introduced some bias as respondents might have feared that giving out information threatened confidentiality. Nevertheless, the reliability of the data derived from NHSS can be assured by the high matching rates of the key indicators between the first and second visits to the 5% households participated the surveys.

## Results

As noted above, this paper seeks to understand the impact of the 1997 urban health insurance reform on insurance coverage. Particular attention is given to the three government-run health insurance schemes (namely GIS, LIS and BHIS). These three government-run schemes will be referred to as mainstream health insurance schemes in the following text and tables.

### Population coverage

From the data displayed in Table 3, BHIS is the most dominant scheme among the insured population, indicating an almost complete transition from GIS and LIS to BHIS between 1998 and 2003. In 2003 total coverage by BHIS was 30.4% (95% CI: 29.8%–31.0%), and only 4.0% (95% CI: 3.1%–4.8%) by GIS and 4.6% (95% CI: 3.7%–5.4%) by LIS. However, the total coverage rates of these three health insurance schemes decreased from 44.7% (95% CI: 44.1%–45.3%) in 1998 to 39.0% (95% CI: 38.3%–39.7%) in 2003 (p < 0.01). One interpretation for this decrease is that dependent children of the insured, who used to be partially covered by LIS, were excluded from BHIS.

**Table 3:** Health insurance coverage for the Urban Population (%), 1998 and 2003

	1998			2003		
	
	Percent	L95%CI	U95%CI	Percent	L95%CI	U95%CI
*Government–run health insurance schemes*						
BHIS	---	---	---	30.4	29.8	31.0
GIS	16.0	15.2	16.8	4.0	3.1	4.8
LIS	28.7	28.1	29.3	4.6	3.7	5.4
MHIS	44.7	44.1	45.3	39.0	38.3	39.7
*Other type of non-commercial insurance schemes*						
Collective schemes	2.7	1.9	3.6	6.6	5.8	7.5
Other	5.1	4.3	6.0	4.0	3.1	4.8
Sub total	7.8	7.1	8.7	10.6	9.8	11.5
Commercial insurance schemes	3.3	2.5	4.1	5.6	4.6	6.3
No insurance cover	44.1	43.5	44.8	44.8	44.2	45.4

* BHIS not existent at the time of the 1998 survey.

Both non-commercial and commercial schemes have increased their share of the insured population between 1998 and 2003. The urban population covered by non-commercial insurance schemes increased from 7.8% in 1998 to 10.6% in 2003, while commercial schemes rose from 3.3% to 5.6% in the same period. Despite the increase, however, there has been little change to the overall figures for the insured and uninsured amongst the total surveyed urban population. In addition, the proportion of the urban population without any insurance cover increased slightly between 1998 and 2003. This means that nearly half of the urban population surveyed had no insurance protection at all against illness and injuries.

### Age

Table 4 shows the percentage in each age group (vertical axis) covered or not covered by health insurance (horizontal axis). The table shows a definite age disparity of participation in health insurance schemes. Older age groups are more like to be covered by mainstream health insurance schemes than younger groups, a tendency also evident in 1998. Nevertheless, the figures show important changes. As BHIS no longer offers any coverage for family dependents, a significant decrease in the population covered by mainstream health insurance amongst the young age groups was seen between 1998 [21.1% (95% CI: 19.2%–23.0%) of the 0–14 group and 24.6% (95% CI: 22.5%–26.6%) of the 15–24 group] and 2003 [5.4% (95% CI:3.1%–7.7%) and 12.3%(95% CI: 9.8%–14.7%) respectively]. However, the increased coverage of non-commercial and commercial health insurance schemes among these two age groups was noticeable between 1998 and 2003.

**Table 4:** Health insurance coverage among age groups (%), 1998 and 2003

	BHIS (1)			GIS+LIS (2)			MHIS (1+2)			Other non-commercial			Commercial			No-insurance cover		
	Percent	95%CI		Percent	95%CI		Percent	95%CI		Percent	95%CI		Percent	95%CI		Percent	95%CI	
												
		L	U		L	U		L	U		L	U		L	U		L	U

*1998*																		
0–14	--	--	--	21.1	19.2	23.0	21.1	19.2	23.0	12.7	10.7	14.6	2.4	0.3	4.4	63.9	62.6	65.1
15–24	--	--	--	24.6	22.5	26.6	24.6	22.5	26.6	10.2	80.	12.5	2.9	0.5	5.2	62.3	60.8	63.8
25–34	--	--	--	42.8	41.2	44.4	42.8	41.2	44.4	7.1	5.1	9.1	4.2	2.2	6.2	45.9	44.4	47.4
35–44	--	--	--	50.8	49.4	52.1	50.8	49.4	52.1	6.8	4.9	8.7	3.4	1.4	5.3	39.1	37.6	40.6
45–54	--	--	--	57.8	56.2	59.3	57.8	56.2	59.3	6.2	3.9	8.4	4.1	1.8	6.4	32.0	30.1	33.9
55–64	--	--	--	62.3	60.7	63.8	62.3	60.7	63.8	5.5	3.0	7.9	3.4	1.0	5.9	28.8	26.7	30.9
65+	--	--	--	58.7	57.2	60.2	58.7	57.2	60.2	6.0	3.7	8.3	2.5	0.2	4.8	32.8	30.9	34.7
Total	--	--	--	44.7	44.1	45.3	44.7	44.1	45.3	7.8	7.1	8.7	3.3	2.5	4.1	44.1	43.5	44.8
*2003*																		
0–14	3.3	0.9	5.7	2.1	1.7	2.4	5.4	3.1	7.7	17.1	14.9	19.3	20.6	18.5	22.7	56.9	55.4	58.5
15–24	9.0	6.5	11.5	3.3	0.7	5.8	12.3	9.8	14.7	16.4	14.1	18.8	10.0	7.6	12.5	61.3	59.6	62.9
25–34	32.0	30.1	33.9	7.1	4.9	9.3	39.1	37.3	40.9	9.4	7.2	11.5	3.4	1.1	5.6	48.2	46.5	49.6
35–44	34.6	32.9	36.3	7.6	5.6	9.6	42.2	40.6	43.8	9.1	7.1	11.0	3.1	1.0	5.1	45.7	44.1	47.2
45–54	40.1	36.5	41.7	10.7	8.7	12.7	50.8	49.3	52.3	9.8	7.8	11.8	2.3	0.2	4.4	37.1	35.5	38.8
55–64	46.6	44.7	48.6	13.2	107	15.7	59.8	58.1	61.5	7.3	4.7	9.8	0.7	0.4	0.9	32.2	30.0	34.5
65+	42.5	40.7	44.2	15.8	13.7	18.0	58.3	56.8	59.8	7.3	5.1	9.6	0.4	0.3	0.5	34.0	32.1	35.9
Total	30.4	29.8	31.0	8.6	7.8	9.4	39.0	38.3	39.7	10.6	9.8	11.5	5.6	4.6	6.3	44.8	44.2	45.4

* BHIS not existent at the time of the 1998 survey.

Although the interpretation of the data in the table needs to take into account population increases in the different groups, it is noticeable that the percentage coverage of mainstream insurance falls for all age groups in the five year period. The age groups 0–14 and 15–24 increased their overall insurance coverage in the period (p < 0.01), while the other five age groups all showed a percentage increase in those without any insurance coverage.

### Employment status

The mainstream insurance schemes are all employment-based and the BHIS operates for both the public and private sectors. However, being in employment does not lead automatically to participation in an insurance scheme. Table 5 relates employment and retirement with participation or non-participation in insurance schemes. It shows that in both 1998 and 2003 slightly over half of the employed population were covered by mainstream health insurance schemes, while about one-third within this employed group were not covered by any insurance arrangements. Corresponding with the findings in the previous section, the retired population were better protected by health insurance, which increased to 74.5% (95% CI: 72.6%–76.4%) of the retired population in 2003 (p < 0.01). In contrast, 75.7% (95% CI: 74.7%–76.6%) and 77.0% (95% CI: 76.1%–77.9%) of individuals not in employment had no health insurance in 1998 and 2003 respectively (p < 0.05).

**Table 5:** Health insurance coverage and employment/retirement status, 1998 and 2003 (%)

	Employed			Retired			Not in employment*		
	
	Percent	95%CI		Percent	95%CI		Percent	95%CI	
						
		L	U		L	U		L	U
*1998*									
BHIS (1)	--	--	--	--	--	--	--	--	--
GIS+LIS (2)	56.1	55.2	57.0	74.1	73.2	75.1	14.1	12.3	16.0
MHIS (1+2)	56.1	55.2	57.0	74.1	73.2	75.1	14.1	12.3	16.0
Other non-commercial	7.3	6.0	8.6	3.3	1.5	5.1	9.4	7.5	11.3
Commercial	4.8	3.5	6.1	3.3	1.5	5.1	0.8	0.6	1.0
No-insurance cover	31.8	30.8	32.9	19.2	17.5	20.9	75.7	74.7	76.6
*2003*									
BHIS (1)	42.9	41.7	43.9	55.3	54.1	56.6	8.4	6.5	10.2
GIS+LIS (2)	10.4	9.1	11.8	18.2	16.5	19.9	1.2	1.0	1.4
MHIS (1+2)	53.3	52.3	54.2	74.5	72.6	76.4	9.6	7.7	11.4
Other non-commercial	10.9	9.6	12.3	5.6	3.1	6.8	10.3	8.5	12.1
Commercial	2.6	1.2	4.0	0.7	0.5	0.8	3.0	1.1	4.9
No-insurance cover	33.1	32.0	34.3	20.7	19.0	22.4	77.0	76.1	77.9

*including not working, unemployed, semi-unemployed

### Income level

Table 6 relates income level/group (1 = lowest income, 5 = highest income level) and participation or not in mainstream insurance schemes. There was a clear correspondence between income level and participation rate amongst the insured: the higher income level, the higher participation rate. Coverage through mainstream insurance fell from 19.7% (1998, 95% CI: 18.0%–21.3%) to 10.7% (2003, 95% CI: 8.9%–12.5%) for income level 1. Comparing the figures for 1998 and 2003, the decline in the participation rate of mainstream health insurance schemes among the income levels 1 to 3 seems to be more severe than those among income levels 4 and 5. The same adverse relation between income level and insurance participation rate was identified by Gao et al [15] in comparing the data from the first two NHSSs in 1993 and 1998, indicating that the declining insurance participation rate amongst the low income population has not been reversed in the past 10 years.

**Table 6:** Health insurance coverage and per capita annual household income and (%), 1998 and 2003

	MIHIS			Other non-commercial			Commercial			No-insurance cover		
	
	Percent	95%CI		Percent	95%CI		Percent	95%CI		Percent	95%CI	
								
		L	U		L	U		L	U		L	U
*1998*												
Lowest	19.7	18.0	21.3	7.6	5.8	9.4	0.9	0.7	1.0	71.9	7.09	72.9
Low	37.1	35.5	38.7	7.3	5.3	9.3	2.0	1.7	2.3	53.6	52.2	55.1
Middle	46.7	45.4	47.9	7.4	5.8	9.1	3.5	1.8	5.2	42.4	41.1	43.7
Higher	56.0	54.7	57.4	8.2	6.2	10.1	5.0	3.1	7.0	30.8	29.1	32.5
Highest	61.8	60.7	62.7	8.8	7.1	10.5	4.8	3.1	6.5	24.6	23.1	26.1
*2003*												
Lowest	10.7	8.9	12.5	7.9	6.0	9.8	4.9	3.0	6.8	76.1	75.1	77.1
Low	26.9	25.2	28.6	12.4	10.5	14.2	5.2	3.3	7.1	55.1	53.8	56.4
Middle	40.7	39.3	42.1	12.2	10.4	13.9	5.4	3.6	7.2	41.1	39.6	42.5
Higher	54.2	52.8	55.6	10.3	8.3	12.3	6.2	4.2	8.2	28.6	26.9	30.4
Highest	63.7	62.5	64.9	10.3	8.4	12.2	5.9	3.9	7.8	19.5	17.7	21.3

All income levels experienced increases in coverage by both non-commercial and commercial health insurance schemes between 1998 and 2003. Although income group 1 showed only a marginal increase in non-commercial health insurance coverage between 1998 and 2003, in comparison with other income groups, the group showed a more significant increase in commercial insurance coverage in the same period.

### Gender

Table 7 shows that males are more likely to participate in mainstream health insurance schemes than females [41.8% (95% CI: 40.8%–42.7%) in 1998 compared with. 35.9% (95% CI: 35.0%–36.9%)] in 2003. This overall trend of gender bias remained almost the same between 1998 and 2003. This is mainly because women are less likely to be in employment than men in China since the economic reform.

**Table 7:** Health insurance coverage and gender (%): 1998 and 2003

	MIHIS			Other non-commercial			Commercial			No-insurance cover		
	
	Percent	95%CI		Percent	95%CI		Percent	95%CI		Percent	95%CI	
								
		L	U		L	U		L	U		L	U
*1998*												
Male	46.8	46.0	47.7	7.7	6.5	8.8	3.6	2.4	4.8	41.9	41.0	42.8
Female	42.7	41.8	43.6	8.0	6.9	9.2	3.0	1.8	4.1	46.3	45.4	47.2
*2003*												
Male	41.8	40.8	42.7	10.4	9.2	11.6	5.5	4.3	6.7	41.8	40.8	42.7
Female	35.9	35.0	36.9	11.0	9.7	12.1	5.5	4.3	6.7	47.1	46.2	48.0

Other types of non-governmental insurance schemes, however, show more balanced gender participation with little change over time.

## Discussion

The data indicated in this paper shows that the social health insurance system is not meeting one of its key aims, that of increasing coverage. Coverage through mainstream insurance has actually decreased and the increase in commercial and other non-commercial insurance fails to increase overall coverage. Although the data from tables 3 to 7 show complex processes of change, it does also suggest concerns regarding the insurance coverage among younger age groups, those not in employment, those on lower incomes and women. Any attempt to meet these concerns requires policies to be based on a comprehensive and in-depth explanation of all these trends and features of coverage. This clearly goes beyond the format of this short paper. However, the discussion can point to three key contextual and policy features that have to be taken into account in any such explanatory analysis: the organisational diversity within the health system; the labour market and insurance coverage; and the growth in non-mainstream insurance coverage.

### Diversity and insurance coverage

At first sight, the merging of the two older insurance systems into the new BHIS appears to seek a new integration and consolidation of the system. The third NHSS showed that 55.2% of the urban population were covered by a number of health insurance schemes, within which BHIS was the most dominant. The merger of the GIS and LIS into BHIS has certainly gone ahead and the previous mainstream systems are now minority insurers. A closer look at the emerging system however, suggests a continuation and deepening of the diversity in insurance systems.

Firstly, there has been a fall in mainstream health insurance which has occurred along with increased diversity in the insurance sector. The non-state insurance sector has expanded. Although there has been a transfer and integration of two old schemes (GIS and LIS) to the new one (BHIS), the transformation is still not complete and GIS and LIS remain in operation. For example, some central government agencies and several provincial government agencies have not participated in the BHIS simply because government civil servants would prefer to continue to enjoy almost free medical care under GIS. This adds to the appearance of diversity in the insurance system. A key issue here is the extent to which the reform does or does not promote risk pooling and achieve cross-group subsidy. Although all enterprises are required to cover the health insurance needs of their employees, participation in the new BHIS is more voluntary, rather than mandatory. Enterprises have the option to take part or stay away, based on their judgement of economic gains/losses. In a study conducted in Shandong province, Meng et al [13] found that the enterprises with a higher proportion of permanent job-holders, retirees, staff of older age, and a history of high medical expenditure were more likely to join the new scheme, whereas the enterprises with staff of a younger age profile and of prosperity tended to look after their staff's medical costs by themselves, or go else where for insurance arrangement. The study [13] pointed out:

"Focus group discussions with enterprise managers and employees revealed that they assessed potential benefits and costs when deciding whether to join the municipal health insurance scheme."

Some enterprises had not yet decided to join the BHIS as they just could not afford the payment of premiums for their employees and retirees. Others thought that it would be better to remain with LIS for the reasons related to financial contributions and benefit packages [13]. Interestingly, a municipal reform of the system in mid 2000 led to an increase in enrolment in the BHIS (idem).

The insurance reform has increased the risk pooling capacity to a higher degree, i.e. from individual enterprise-based to municipal-wide. In spite of the increased pooling size, the new scheme has not achieved a satisfactory level of solidarity. In fact, like the old schemes, the new scheme has maintained more or less the same eligibility criteria based on employment. This can potentially reduce the basis for wider horizontal and vertical subsidy between the rich and poor, young and old, and the sick and healthy.

Secondly, BHIS operates through a decentralised system in which local government plays an important part. Although there is a national framework for the operation of the BHIS, it also operates through a decentralised system in which local government plays a role in setting the rules and regulations of the system. On the one hand, this decentralisation of the system can have an impact on service provision and lead, for example, to different staff salary and benefits packages in different districts/regions at different stages of economic development [19]. On the other hand, different local regulations on the operation of the public insurance system can impact on coverage. Both factors can lead to inequity in the operation of the health system.

### Labour force policy and insurance coverage

The economic reform from the 1980s onwards has brought with it a rapid process of economic growth and urbanisation in China. Major changes have taken place in the mechanisms and composition of the labour market. The selectiveness of the BHIS may be closely linked to these changes.

Firstly, since the economic reform in the 1980s, enterprises have been given more autonomy in managing their staff. For example, increasing numbers of staff have been employed on short-term contracts [20]. Government regulation requires all companies to buy health insurance for their contracted staff. However, the implementation of such regulations may vary a great deal among these institutions. Staff on short-term contracts may have 'fewer benefits' than other staff [21] while Tolhurst et al [22] argue that:

"...discrimination in the labour market against women (...) reduces their health insurance entitlements, either because schemes are unable to offer benefits to laid – off or early retired workers or because individuals on short term or temporary contracts have reduced insurance entitlements."

Secondly, rapid economic expansion in urban areas has created massive rural to urban movement in the labour force. Consequently this has formed a new population group of non-registered migrants residing in urban areas [20]. According to the new statistics, 114 million rural migrants were estimated to be living in the urban cities of China [23]. As Bloom [21] and Meng et al [13] commented, urban household registration can be important in securing access to health care, although the system of registration is under reform. Meng et al [13] point out:

"The precise number of this 'floating' population is uncertain, but is thought to be at least double estimates. This population is not covered by any social insurance system. Whether they have any insurance status at all is determined solely by their employer, if they have been fortunate enough to secure a job."

Thirdly, the gender differences in insurance coverage need to be understood in the context of gender differences in employment. This study showed that the urban female population in China are distinctly disadvantaged in insurance coverage. Given that the BHIS is employment-based, this may be looked at from the angle of the employment status of women in urban areas. In 2002, only 38% of total employees in urban units were female [24]. In spite of the political dedication to and promotion of women's involvement in economic activities since 1949, economic reform and retrenchment of the work force has undermined women's employability to a large degree [25]. Gender bias over mainstream insurance participation have been reported by, for example, Gao et al [15] and may be traced to the gender biased employment patterns. In the advent of the market economy and the relaxation of workforce policies, competition for employment that offers better pay and/or benefits becomes increasingly fierce. More frequently, women, particularly older women, find themselves disadvantaged in the labour market in comparison with their male counterparts [22].

Fourthly, the mainstream health insurance schemes are, more accurately, institutional and employment-based and therefore do not cover the self-employed. This rule does not seem to coincide with the changing employment features in urban areas. From 1996 to 2000, people in self-employment in urban settings increased from 17.09 to 21.36 million [17]. Although it is recognised that assessing the annual income of the individuals in this group may pose difficulties [26], some municipal governments, such as in the city of Guiyang and Urumuqi Autonomous Region, have allowed the self-employed to participate in BHIS, as long as they are willing to pay the premium which ranges between 5–8% of the average annual salary of the city employees. However, for the self-employed, the contribution to the premium is solely from the insured and there is no subsidy from government.

Fifthly, unemployment in urban areas increased from 3.1% in 2000 [17] to 4% in 2002 [27], although the actual unemployment rate could be much higher than these figures as many unemployed do not register their employment status with the government agencies [27]. The unemployed are clearly not covered by any mainstream health insurance schemes. Although economic enterprises do have the legal obligation to arrange benefits, including health insurance, for 'laid-off' workers [16], the implementation of this policy has not been effectively enforced due to a number of reasons, particularly the poor financial status of many enterprises [1].

Lastly, this paper also points to an imbalance in the relationship between the employer/owner of the enterprises and their employees. We have already noted that the process of economic reform from the 1980s onwards, owners/employers have been granted increased management authority in the running of enterprises. There is no guarantee that this authority is being used in the interests of the employees. At the same time, trade unions and workers congresses in China exert little influence in industrial relations and decision making on workers' benefits including health insurance coverage. This is largely due to their poor capacity in lobbying for policy change within and across enterprises, and the reluctance of and resistance from government and enterprises to granting trade unions independent status in representing workers [28,29]. Against this background, mandatory requirement for participation in BHIS may need to be examined in a wider context of strengthening the law to protect employees'/workers' rights and entitlements.

### Non-mainstream forms of insurance schemes

As noted above, the NHSSs revealed the growth of urban population coverage by other types of health insurance schemes outside the mainstream insurance schemes. The commercial insurance schemes are of recent origin and may well continue to grow as part of a general shift to private forms of production and exchange in China. The 2003 NHSS data showed that the coverage rate by commercial insurance varied a great deal between cities [16], probably depending on the establishment and acceptability of commercial insurance, and availability and accessibility of mainstream health insurance and other non-commercial insurance schemes. However, discriminatory selection criteria, limited coverage of health care needs and high premium rates [30] raise the question as to whether these schemes can meet fully the health care needs of vulnerable groups such as the elderly and low income families.

The non-commercial (and non-mainstream) medical insurance schemes are diverse and complex. They are largely made up of non-profit-making arrangements set up by different government departments, public institutions, or enterprises to cover certain medical care needs of different groups. Included here are Medicaid schemes for the urban poor, catastrophic disease insurance schemes, collective health insurance, insurance arrangements made by schools or enterprises for students/employees, as well as rural collective medical insurance by migrant workers working in urban areas [30]. There is, however, little literature on the subject although there is a huge variation in the establishment and population coverage rates for catastrophic disease insurance schemes and cooperative health insurance schemes between cities [16]. Both these types of schemes are run by local governments, and the variation reflects, once again, the interrelationship between health insurance policy and local contexts.

Recent policy development and discussion has turned to the issue of integrating non-commercial health insurance schemes into the mainstream medical insurance scheme, or broadening the eligibility for mainstream health insurance schemes to uninsured vulnerable groups. The policy document issued by the State Council in 2006 stated that eligible migrant workers should be included into basic medical insurance schemes [31]. In Shanghai the municipal government recently announced that eligible uninsured elderly (70+ years) can participate in the mainstream insurance schemes managed by the municipal medical insurance agency [32]. School children and university students have also been included in basic health insurance schemes in some cities [33,34]. However, the reinforcement of the policies that address health insurance for vulnerable groups will depend on the mandatory status of these policies and the capacity of local governments to implement them. This is, of course, little different from the problem faced by BHIS, as discussed earlier.

Further research is needed to compare the different insurance schemes currently operating in China in terms of, for example, financial protection, service/benefit coverage, and levels of acceptability and satisfaction among the insured. This research should take into account the contextual settings in which the different schemes have been established and operated. It is important that such research should inform the further developments of social health insurance policy in China. Further policy relevant research is also needed into how different options of insurance administration impact on efficiency. For example, merging different non-commercial health insurance schemes and including dependent children and spouses into employees' insurance scheme could be analysed.

## Conclusion

Based on a comparison between two NHSSs, this paper has drawn attention to the changing face of health insurance in China. In addition to the increasing diversity in insurance systems, it has also drawn attention to the growing inequity in insurance coverage between 1998 and 2003. The rapid economic development and the reduction in poverty that has taken place in recent years have contrasted with restrictions in coverage and critical issues around the inequity generated within the system.

Unless appropriate action is taken, it is quite possible that the forms of gender, age, income and employment disparities indicated will continue to increase. The discussion suggests the need to look at state involvement in two possible areas. Firstly, there is a need to consider establishing and managing insurance schemes particularly for the vulnerable groups, such as low-income and short-contract employees. The development of public insurance schemes benefiting disadvantaged groups including women and the poor in urban settings, such as the medical finance assistance scheme, is still in its infancy. They have only been piloted in a handful cities [1], although the Ministry of Civil Affairs wants to move ahead quickly in this area.

Secondly, there is the option of legislation and regulatory control to widen access to joining the BHIS. As the implementation of the new health insurance scheme has not been made mandatory for all the institutions and enterprises [1], selective coverage is still a major feature of the new health insurance scheme. Measures including interim policies need to be considered to make the new scheme more inclusive, particularly for the vulnerable groups.

There is clearly a need for research to monitor and evaluate the impact of new forms of health insurance. Results analysed in this paper are certainly cause for concern. In conducting research, this paper suggests four important characteristics to be taken into account. Firstly, the research has to cover the broader aspects of health insurance systems, going beyond the subject matter of this paper. For example, further work needs to be done in such vital areas as financing and the relative impact financial changes place on different groups and changes in benefit coverage. It would be useful to assess and compare the level of benefit coverage and financial burden on different groups under different health insurance schemes including BHIS, the old systems (GIS-LIS) and other non-commercial schemes. The impact on member access to health care in the light of the different forms of user charging and the individualised nature of the insurance accounts needs to be understood. Secondly, the data in this paper is aggregated to represent national trends. On several occasions this paper has pointed to the regional and local diversities to be found in the new system. It is important that research takes up this challenge of demonstrating how differences in both the regulatory norms of the insurance systems and the differences in the local economic and social contexts can have an impact on the outcome of insurance reform. It is quite possible that the decentralised system of BHIS insurance management is a step to further inequity. Thirdly, the research needs to recognise the complexity in identifying causal relations between insurance reform and impact on health care. There are a wide range of contextual and contradictory factors determining the type and level of health care provided to different social groups. Insurance reform is one among a wide range of factors to consider. Research needs to unravel how insurance reform relates to these other factors and impacts on such factors as the type of care and the level of equity. Lastly, further research needs to integrate both qualitative and qualitative methods. This is important for developing complex patters of causal explanations between insurance reform and such issues as coverage, access and cost containment.

## Competing interests

The authors declare that they have no competing interests.

## Authors' contributions

**LX **contributed significantly to the conception of the paper, analysis and interpretation of data, and participated in revising the paper for important intellectual content, also participated in the conduct and management of the National Health Service Survey in 2003;

**YW **contributed significantly to the conception and drafts of the paper, analysing and interpreting data, and revising the paper;

**CDC **carried out revising the paper critically for important intellectual content and gave the final approval of the version to be published;

**ST **contributed to the conception of the paper, data analysis, and revising it critically for important intellectual content.

All the authors read and approved the final manuscript.

## Pre-publication history

The pre-publication history for this paper can be accessed here:



## References

[B1] Tang S, Liang H, Meng Q, Baris E (2003). Addressing inequity in access to health care in urban China: a review of health care financing reform experiment. Working Paper Series NO 2004-5.

[B2] Liu Y (2002). Reforming China's urban health insurance system. Health Policy.

[B3] Ministry of Labour and Social Security Statistic report of labour and social security development for 2004 by Ministry of Labour and Social Security, China. http://www.molss.gov.cn/gb/zwxx/2005-12/14/content_99533.htm.

[B4] Palmer N, Mueller DH, Gilson L, Mills A, Haines A (2004). Health financing to promote access in low income settings--how much do we know?. The Lancet.

[B5] WHO (2000). The World Health Report 2000 - Health Systems: improving performance.

[B6] WHO (2005). The world health report 2005: make every mother and child count.

[B7] Tang S, Meng Q, Bloom G, Tang S (2004). Introduction to the urban health system and review of reform initiatives. Health care transition in urban China.

[B8] The World Bank (1997). Financing health care: issues and options for China.

[B9] Liu X, Hsiao W (1995). The cost escalation of social health insurance plans in China: its implication for public policy. Social Science & Medicine.

[B10] Henderson G, Jin S, Akin J, Li Z, Wang J, Ma H, He Y, Zhang X, Chang Y, Ge K (1995). Distribution of medical insurance in China. Social Science and Medicine.

[B11] Hu TW, Ong M, Lin ZH, Li E (1999). The effects of economic reform on health insurance and the financial burden for urban workers in China. Health Economics.

[B12] Hsiao W, Dunlop DW, Martins JM (1995). A framework for assessing health financing strategies and the role of health insurance. An international assessment of health care financing.

[B13] Meng Q, Weng Z, Zhuang N, Wang W, Sun Q, Lu Y, Shu B, Bloom G, Bloom G, Tang S (2004). The health systems of Nantong and Zibo. Health care transition in urban China.

[B14] Ministry of Health of China (1998). The design and guidance for the second National Health Services Survey.

[B15] Gao J, Tang S, Tolhurst R, Rao K (2001). Changing access to health services in urban China: implication for equity. Health Policy and Planning.

[B16] Centre for Health Statistics and Information MH (2004). An analysis report of National Health Services Survey in 2003.

[B17] National Bureau of Statistics of China (2001). China Statistical Yearbook 2001.

[B18] Ministry of Health of China (1999). Research on National Health Services ---an analysis report of the second National Health Services Survey in 1998 (I).

[B19] Wang Y, Collins C, Tang S, Martineau T (2004). Health system decentralization and human resources management in low and middle income countries. Public Administration and Development.

[B20] Bloom G, Lu Y, Chen J (2002). Financing health care in China's cities: balancing needs and entitlements during rapid change.

[B21] Bloom G, Bloom G, Tang S (2004). China in transition: challenges to urban health services. Health care transition in urban China.

[B22] Tolhurst R, Standing H, Qian X, Bloom G, Tang S (2004). Gendered impacts and implications of health sector reform in the context of multiple transitions in urban China. Health care transition in urban China.

[B23] Xinhua News Agent China had 113.9 million migrant workers in 2003. http://www.chinadaily.com.cn/english/doc/2004-05/15/content_330991.htm.

[B24] Information Office of State Council Labour and social security in China by Information Office of State Council. http://www.china.org.cn/e-white/20020429/index.htm.

[B25] Appleton S, Knight J, Song L, Xia Q (2002). Labour retrenchment in China: Determinants and consequences. China Economic Review.

[B26] McPake B, Kumaranayake L, Normand C (2002). Health economics: an international perspective.

[B27] National Bureau of Statistics of China Women and Men in China by National Bureau of Statistics of China. http://www.stats.gov.cn/tjsj/qtsj/men&women/men&women.pdf.

[B28] Ding DZ, Goodall K, Warner M (2002). The impact of economic reform on the role of trade unions in Chinese enterprises. International Journal of Human Resource Management.

[B29] Shen J (2006). An analysis of changing industrial relations in China. The International Journal of Comparative Labour Law and Industrial Relations.

[B30] Wu M, Centre for Health Statistics and Information (2004). Study on health service utilisation and barriers among rural migrant workers in Beijing. Research on health reform issues in China, 2003.

[B31] Ministry of Labour and Social Security On widening migrant workers' participation in health insurance schemes. http://trs.molss.gov.cn/was40/mainframe.htm.

[B32] Policy document issued by Shanghai Health Insurance Bureau and Shanghai Labour and Social Security Bureau on uninsured elderly participating basic health insurance schemes. http://www.law-lib.com/law/law_view.asp?id=171662.

[B33] Shanghai Health Insurance Bureau Most asked questions on health insurance. http://ybj.sh.gov.cn/faq/faq_main.jsp.

[B34] The students in the City of Qiqihaer to be covered by Basic Health Insurance. http://news.xinhuanet.com/edu/2006-06/24/content_4742151.htm.

